# Early-life tobacco smoke exposure and stroke risk: a prospective study of 341,783 and 352,737 UK Biobank participants

**DOI:** 10.1186/s12889-024-18588-6

**Published:** 2024-05-17

**Authors:** Fabin Lin, Xuanjie Chen, Yisen Shi, Kaitai Yang, Guoping Hu, Weijiang Zhuang, Yifei Lin, Tingting Huang, Qinyong Ye, Guoen Cai, Xilin Wu

**Affiliations:** 1 Department of Neurology, Center for Cognitive Neurology, Institute of Clinical Neurology, Fujian Medical University Union Hospital, 29 Xinquan Road, 350001 Fuzhou, China; 2 Fujian Institute of Geriatrics, Fujian Medical University Union Hospital, 29 Xinquan Road, 350001 Fuzhou, China; 3 Fujian Key Laboratory of Molecular Neurology, Fujian Medical University, 88 Jiaotong Road, 350001 Fuzhou, China; 4 Fujian Medical University, Fuzhou, China; 5 Department of Neurosurgery, Fujian Medical University Union Hospital, 29 Xinquan Road, 350001 Fuzhou, China

**Keywords:** Stroke, Tobacco, Early-life, Cohort

## Abstract

**Introduction:**

Stroke is a life-threatening condition that causes a major medical burden globally. The currently used methods for the prevention or prediction of stroke have certain limitations. Exposure to tobacco in early life, including smoking during adolescence and maternal smoking during pregnancy, can affect adolescent development and lead to several negative outcomes. However, the association between early-life tobacco exposure and stroke is not known.

**Methods:**

In this prospective cohort study, for the analyses involving exposure to maternal smoking during pregnancy and age of smoking initiation, we included 304,984 and 342,893 participants, respectively., respectively from the UK Biobank. Cox proportional hazard regression model and subgroup analyses were performed to investigate the association between early-life tobacco exposure and stroke. Mediation analyses were performed to identify the mediating role of biological aging in the association between early tobacco exposure and stroke.

**Results:**

Compared with participants whose mothers did not smoke during pregnancy, participants whose mothers smoked during pregnancy showed an 11% increased risk of stroke (HR: 1.11, 95% CI: 1.05–1.18, *P* < 0.001). Compared with participants who never smoked, participants who smoked during adulthood, adolescence and childhood showed a 22%, 24%, and 38% increased risk of stroke during their adulthood, respectively. Mediation analysis indicated that early-life tobacco exposure can cause stroke by increasing biological aging.

**Conclusion:**

This study reveals that exposure to tobacco during early life is associated with an increased risk of experiencing a stroke, and increased biological aging can be the underlying mechanism.

**Supplementary Information:**

The online version contains supplementary material available at 10.1186/s12889-024-18588-6.

## Highlights

The first study to investigate the association between early-life tobacco exposure and stroke in a large-scale cohort database.

The significant association between early-life tobacco exposure and the occurrence of stroke has been observed.

Accelerated biological aging acts as a mediator in the association between early-life tobacco exposure and stroke.

**Supplementary Information:**

The online version contains supplementary material available at 10.1186/s12889-024-18588-6.

## Introduction

Stroke is a leading cause of long-term disability and accounts for approximately 10% of global mortality. Stroke-associated mortality has increased over the past two decades, contributing to the increased burden of stroke globally [1–3]. Therefore, it is important to identify the risk factors of stroke and implement preventive strategies.

Tobacco is known to contain several harmful compounds and exposure to tobacco smoke can contribute to the risk of stroke [4–6]. However, most studies have focused on smoking habits in adults [4, 7], and the effect of exposure to tobacco smoke during early life on the risk of stroke is limited.

Previous studies have reported that exposure to tobacco smoke during early life is associated with an increased risk of several diseases during adulthood [8–10], which can affect developmental processes and cause long-term health problems [11].. Moreover, early-life tobacco exposure can affect the immune system and telomere length [12, 13]. Some early-life factors, including socioeconomic status, exposure to famine, and other factors, can increase the risk of stroke [14–16]. Early-life tobacco smoke exposure includes maternal smoking during pregnancy and the age at which an individual starts smoking. Nevertheless, the association between early-life tobacco smoke exposure and the risk of stroke is ambiguous. Previous studies have indicated that smoking may cause telomere damage, leading to cellular aging [17]. Furthermore, biological age has been suggested to be influenced by various risk factors across multiple subtypes [18, 19].

In this prospective cohort study, we obtained data from the UK Biobank, with substantial sample size and comprehensive information, to investigate the association between early-life exposure to tobacco smoke and the risk of stroke. We also elucidated the mechanism underlying the role of biological age in this association.

## Methods

### Data source and study participants

The UK Biobank is a substantial, prospective epidemiological study that aims to explore the role of general exposure in healthcare and disease. The UK Biobank enlisted approximately 500,000 participants ranging in age from 37 to 73 years between 2006 and 2010, gathered from 22 assessment centers across the UK. The enrolled participants filled out touchscreen questionnaires, underwent physical measurements, and submitted biological samples. Participants were tracked via links to national health records and follow-up visits to develop event diagnoses. The UK Biobank obtained approval from the National Research Ethics Service under the reference number 11/NW/0382. Studies involving human participants were reviewed. Each patient/participant was provided with written informed consent [20, 21].

All covariates, exposures, and outcome data for all participants in the final study sample need to be complete without any missing values. In the end, the final sample sizes for exposure to maternal smoking during pregnancy and age of smoking initiation were 304,984 and 342,893, respectively (Figure.S1).

### Early-life exposure to tobacco smoke

In the UK Biobank, early tobacco exposure is evaluated based on the following two components: the age at which participants started smoking and whether their mothers smoked during pregnancy. For participants who regularly smoke or those with a history of smoking, the age of initiation was determined through the “age started smoking in current smokers” (Field ID: 3436) and “age started smoking in former smokers” (Field ID: 2867) variables. To evaluate maternal smoking during pregnancy, the variable “maternal smoking around birth” (Field ID: 1787) was used [22]. The analysis of the age of smoking initiation included data on current smokers and former smokers. The age at smoking initiation was categorized as adulthood (> 18 years), adolescence (15–18 years), childhood (< 15 years), and never smokers.

### Determination of stroke onset

Stroke was identified using algorithmic defined outcomes (ADOs) (Field ID: 42,006) from the UK Biobank. The event sources of ADOs were coded information obtained from the baseline assessment data collection of the UK Biobank, such as participant self-reported medical condition, surgery, and medication data, relevant data from admissions such as diagnoses and procedures, and death registries. The use of ADOs for disease confirmation has been reported in previous studies [23]. These data were algorithmically combined and categorized using UK Biobank Self Report Codes (Code 1081, Code 1086, Code 1491, and Code 1583), ICD 9 Codes (430.X, 431.X, 434.X, 434.0, 434.1, 434.9, and 435.X) and ICD 10 Codes (I60, I60.1–I60.9, I61, I61.0–I61.9, I63, I63.0–63.9, and I64.X). More detailed information about ADOs is mentioned in the UK Biobank [24]. Previous studies have also used this algorithm [23].

### Assessment of biological ages

We have used Klemera-Doubal method Biological Age (KDM-BA) and Phenoage which are recognized algorithms used for detecting the biological age of individuals. As shown in previous studies, these algorithms can be implemented using data obtained from the evaluation of blood parameters of the participants in the UK Biobank [25, 26]. The predicted age by KDM-BA is consistent with the actual age of the individual at which their physiology is considered approximately normal. The KDM-BA is generated via a set of regressions where chronological age is plotted against individual biomarkers within the reference population. The equation uses information obtained from n regression lines, each corresponding to n biomarkers plotted against chronological age. KDM-BA is computed based on forced expiratory volume in one second (FEV1), systolic blood pressure, and seven blood chemistry parameters. The PhenoAge algorithm was established through the multivariate analysis of mortality hazards. The initial PhenoAge algorithm was established by performing an elastic net Gompertz regression, including mortality rates for 42 biomarkers in NHANES III. PhenoAge is calculated based on nine blood parameters, four of which overlap with KDM-BA. Details regarding the corresponding UK Biobank data sources in the algorithm for biological age are shown in Supplemental Table S1. The computation of biological age values was performed using the R package “Bioage,” which serves as a toolkit for the quantification of biological age based on blood chemistry and organ function test data. Further information on this toolkit can be found at BioAge–PMC (nih.gov) (https://github.com/dayoonkwon/bioage). To determine the differences between biologically aged participants, we regressed their calculated biological age values on their actual age during the time of the biomarker measurements and calculated residual values. We refer to these residuals as “age-accelerated (AA)” values to evaluate biological aging.

### Measurement of covariates

Regarding the covariates included in the study, we incorporated gender (male, female), age, race, education level (whether achieved vocational education level), TDI, physical activity, alcohol consumption status (current, previous, never), hypertension (yes, no), and diabetes (yes, no) and healthy diet score. A healthy diet score was calculated by considering the following five factors: consuming at least 4 tablespoons of vegetables per day, eating a minimum of 3 pieces of fruit daily, including fish in the diet at least twice a week, limiting processed meat intake to once a week, and restricting unprocessed red meat consumption to twice a week. Each criterion corresponds to the median value for that specific item. One point was assigned to each satisfied criterion, resulting in a total diet score ranging from 0 to 5. The determination of the healthy diet score was based on previous research [19].

### Statistical analysis

To determine variances in baseline characteristics between groups categorized by early tobacco exposure (age of smoking initiation and exposure to tobacco smoking in utero), we performed chi-square (χ^2^) tests for categorical variables and analysis of variance (ANOVA) for continuous variables. The follow-up duration was determined from the date of attending assessment centre to the occurrence of stroke diagnosis, death, loss to follow-up, or the date of the last admission data in England (September 30, 2021), Scotland (July 31, 2021), and Wales (February 28, 2018), whichever came first. Cox proportional hazard models were used to determine the association between early-life tobacco exposure (age of smoking initiation and Exposure to Tobacco Smoke In Utero) on stroke risk. Furthermore, three models were used for association analysis. Model 1 was not adjusted for covariates. Model 2 was adjusted for age, sex, race, BMI, TDI, physical activity, and alcohol consumption. Model 3 was further adjusted for hypertension, diabetes, and healthy eating scores based on Model 2. Cox proportional risk model results are presented as hazard ratio (HR) and 95% confidence interval (CI). The Schoenfeld residual method was performed to assess the proportional risk assumptions of the Cox model, and all tested assumptions were met.

We performed subgroup analyses to investigate the effect of various subgroup factors (such as gender, hypertension, diabetes, BMI, and age) on the analysis. Likelihood ratio tests were performed to determine the interactions between subgroup variables and early tobacco exposure (such as age of smoking initiation and exposure to tobacco smoke in utero). We performed mediation analyses using the R package “mediation” to investigate whether increased biological aging mediated the association between early smoking exposure and the risk of stroke. We also performed a series of sensitivity analyses. We validated the stability of the results by excluding participants who suffered from stroke or diabetes within 1 year of baseline. We also performed the analyses after excluding participants with diabetes. Furthermore, we created a sample by simultaneously removing participants with missing data on maternal smoking during pregnancy and age of smoking initiation. We then added age of smoking initiation as a covariate in the model where the exposure was maternal smoking during pregnancy, and maternal smoking during pregnancy as a covariate in the model where the exposure was age of smoking initiation. This approach was undertaken to comprehensively consider participants’ complete early tobacco exposure history during analysis. We excluded participants with household smokers from the analysis of age of smoking initiation to eliminate the influence of secondhand smoke on participants. We included “smoking cessation for more than 6 months” as a covariate in a sensitivity analysis to demonstrate that smoking cessation status does not affect the robustness of the results.

## Results

### Characteristics of participants

In the analysis of maternal smoking during pregnancy, we included 341,783 participants, among which 89,254 participants reported that their mothers smoked during pregnancy. In total, 352,737 participants were included in the analysis of age of smoking initiation, among which 233,792 participants reported never having smoked. The majority of participants who started smoking before the age of 18 were female and had a history of diabetes and high blood pressure (Table.S2).

### Exposure to tobacco smoke during early life and stroke risk

The Cox proportional hazards regression analysis showed that participants whose mothers smoked during pregnancy exhibited an 11% higher risk of stroke compared with participants whose mothers did not smoke during pregnancy. (HR: 1.11, 95% CI: 1.05–1.18, *P* < 0.001). Furthermore, compared with non-smokers, participants who started smoking during adulthood, adolescence, and childhood exhibited an increased risk of stroke by 22%, 24%, and 38%, respectively (adulthood, HR: 1.22, 95% CI: 1.14–1.31, *P* < 0.001; adolescence, HR: 1.14, 95% CI: 1.16–1.33, *P* < 0.001; childhood, HR: 1.38, 95% CI: 1.25–1.52, *P* < 0.001) (Table 1).

### Subgroup analysis

The subgroup analysis focusing on maternal smoking during pregnancy showed that the association between maternal smoking during pregnancy and stroke risk was not found in participants with normal and increased blood pressure (normal, HR: 1.05, 95% CI: 0.86–1.29; increased, HR: 1.11, 95% CI: 0.92–1.34). No subgroup factor indicated an interaction with maternal smoking during pregnancy (Table 2).

In the subgroup analysis focusing on the age of smoking initiation, the association between the age of smoking initiation and stroke risk was not found in participants with diabetes, prehypertension, and stage 1 hypertension. Age of smoking initiation exhibited interactions with age (*P* for interaction = 0.045), BMI (*P* for interaction = 0.017), hypertension (*P* for interaction = 0.004), and gender (*P* for interaction = 0.047) at the time of the survey (Table 3).

In the subgroup analysis based on whether mothers smoked during pregnancy, the relationship between the age of smoking initiation and stroke risk was the same. In the group exposed to maternal smoking during pregnancy, smoking during childhood increased the risk of stroke by 42%, while in the group where mothers did not smoke during pregnancy, smoking during childhood increased the risk of stroke by 30%.(Table.S3).

### AA is a mediating factor in the association between age of smoking initiation and stroke risk

**Fig. 1:**
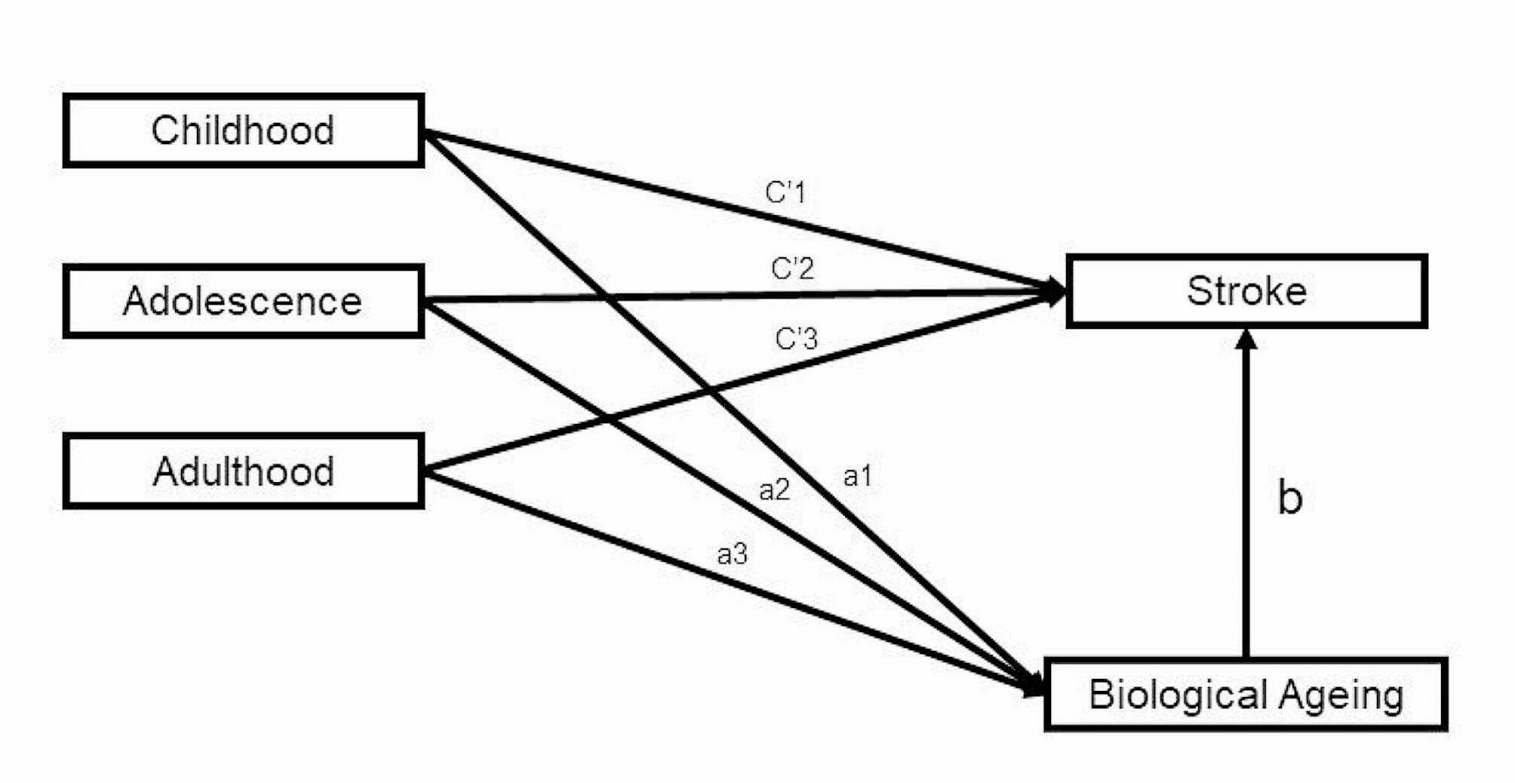
Path diagram of mediation model. “a1-a3”: the effect of age at smoking initiation on biological ageing; “b”: the effect of biological ageing on risk of stroke; “c’1-c’3”: the effect of age at smoking initiation on risk of stroke

The pathway diagram for the mediation model is shown in Fig. 1. PhenoAge AA plays a certain mediating role in the association between age of smoking initiation and stroke occurrence (Indirect Effects, Adulthood = 0.0008, Adolescence = 0.0009, Childhood = 0.0014; Proportion of mediation, Adulthood = 18.34%, Adolescence = 21.70%, Childhood = 25.10%), KDM-BA AA also mediated the association between age of smoking initiation and stroke occurrence (Indirect Effects, Adulthood = 0.0002, Adolescence =; Proportion of mediation, Adulthood = 6.44%, Adolescence = 11.10%, Childhood = 16.64%) (Table 4).

### Sensitivity analysis

We conducted the following series of sensitivity analyses. First, we analyzed the data after excluding individuals who developed the disease within one year after baseline (Table S5), second, we conducted the analysis after excluding individuals with diabetes (Table S6), third, we conducted the analysis using a complete sample and added other covariates related to smoking (Table S7), fourth, we analyzed the data after excluding individuals with household smokers, finally, we conduct the analysis after further adjusted stop-smoking status to the model. All sensitivity analysis results are consistent with the significance observed in previous results.

## Discussion

In this large-scale cohort study, we revealed a significant association between exposure to tobacco smoke during early life and an increased stroke risk. Individuals whose mothers smoked during pregnancy exhibit a higher risk of stroke compared with individuals whose mothers did not smoke during pregnancy. Individuals who started smoking during adulthood, adolescence, and childhood exhibit an increased and progressively higher risk of stroke compared with non-smokers. The age of smoking initiation is associated with accelerated biological aging. Furthermore, increased biological aging acts as a mediator in the association between exposure to tobacco smoke during early life and the occurrence of stroke.

Our findings based on a large cohort study are consistent with previous research [27]. The results of this study provide valuable insights and highlight the effect of early tobacco exposure on the risk of stroke. This study provides a scientific background for the development of appropriate prevention and intervention strategies.

The results of this indicate that early-life tobacco smoke exposure is associated with a higher risk of stroke. Previous studies have focused on the relationship between smoking or secondhand smoke exposure and the risk of stroke [28, 29]. However, the effect of tobacco smoke exposure during early-life stages on stroke is not known. A study based on the Swedish population reported the association between tobacco use and stroke risk in males aged 20 years and below. The study concluded that individuals under the age of 20 who smoke exhibit an increased risk of stroke [30]. However, the study did not differentiate different age groups based on the age of smoking initiation. Exposure to tobacco smoke during early life may lead to changes in enzymes, hormone levels, gene expression, microRNA, and protein expression during the growth and development processes, therefore, it can potentially affect uterine gene expression and disease susceptibility [31]. Early-life tobacco smoke exposure can profoundly affect the immune system of newborns [13, 32]. The changes in the immune system have significant associations with the risk of stroke. The immune system plays a dual role in the pathophysiology of stroke. It can contribute to the damage and exacerbation of brain injury after stroke, which may be targeted as a potential therapeutic approach to mitigate the effect of stroke [33, 34].

Our subgroup analysis showed that the age of smoking initiation has different effects based on age, BMI, hypertension, and gender. Previous studies have also reported that factors such as gender, BMI, hypertension, and age are significant risk factors for stroke, early-life tobacco exposure may exacerbate the impact of these factors on stroke. Two previous studies have shown that age and gender are risk factors for stroke [35, 36]. Furthermore, individuals with obesity and hypertension also have a higher risk of stroke [37, 38]. In the group exposed to maternal smoking during pregnancy, smoking during childhood increased the risk of stroke by 42%, whereas in the group where mothers did not smoke during pregnancy, smoking during childhood increased the risk of stroke by 30%. Therefore, maternal smoking during pregnancy can have an additive effect on the risk of stroke associated with smoking during childhood, thereby increasing the risk of stroke in individuals during adulthood.

Our analysis revealed a noteworthy relationship between the age of smoking initiation and AA, which is consistent with the results of previous research results [39, 40]. The mediation analysis suggests that both the age acceleration calculation methods (KDM-BA AA and PhenoAge AA) serve as mediators in the relationship between the age of smoking initiation and stroke risk. Cigarette smoking-induced cellular damage, structural abnormalities, and decreased cellular regenerative capacity are considered potential mechanisms [41], that increase AA during the same time.

This study has many noteworthy strengths. We used a large and long-term follow-up cohort from the UK Biobank, including a substantial sample size and extending the duration of the analysis. The inclusion of adequate stroke events and early-life tobacco smoke exposure within this dataset improved the statistical analysis. Furthermore, we carefully classified early tobacco exposure into the following three stages: smoking during pregnancy and smoking before the age of 18. This approach allowed us to examine stroke risk among individuals with different tobacco exposure patterns during these different life stages.

This study has certain limitations that should be addressed. First, the specific type of smoking and the frequency and intensity of smoking were not recorded. Therefore, we could not include these aspects in our analysis. Second, our findings may not precisely reflect the complete complexity of smoking patterns and their potential effects on the risk of stroke. Third, the study was performed based on the data obtained from the UK population; hence, the results cannot be generalized to other populations. Fourth, cultural and environmental factors and different healthcare systems can also affect the association between exposure to tobacco smoke during early life and the risk of smoke among diverse populations. Fifth, although we used multiple confounding variables and three models with appropriate adjustments for analysis, certain determinants may remain unmeasured. Hence, the potential for reverse causation cannot be completely ignored. We have taken measures to mitigate these limitations, but further research is warranted to address these limitations comprehensively and improve the robustness of our results. Sixth, our analytical model did not fully account for some additional stroke risk factors such as left atrial enlargement and prevalent cardiovascular diseases. This could potentially introduce bias into the results. Finally, our study population predominantly comprises individuals from the United Kingdom, who typically exhibit higher levels of health and financial status compared to the global average. This could potentially introduce confounding bias into the study. Recall bias exists in this study, which could potentially lead to bias in the results.

## Conclusion

We revealed an association between early-life exposure to tobacco smoke and an increased risk of stroke in life. Mediation analyses indicated that early-life tobacco smoke exposure can accelerate biological age to increase the risk of stroke.

**Table 1:** Association between exposure to tobacco smoke during early life and stroke risk

	In utero tobacco smoke exposure		Age of smoking initiation				
	No	Yes	Never smokers	Adulthood ≥ 18 years	Adolescence 15–18 years	Childhood < 15 years	*P*for trend
Number	215,730	89,254	233,792	16,969	46,612	45,520	
Events	4061	1830	3954	466	1232	1140	
Model 1^a^	1 (Reference)	1.09 (1.03, 1.15) 0.002	1 (Reference)	1.52 (1.42, 1.62) <0.001	1.61 (1.51, 1.71) <0.001	1.69 (1.53, 1.86) <0.001	0.042
Model 2^b^	1 (Reference)	1.12 (1.06, 1.18) <0.001	1 (Reference)	1.24 (1.16, 1.32) <0.001	1.26 (1.18, 1.35) <0.001	1.42 (1.28, 1.56) <0.001	0.033
Model 3^c^	1 (Reference)	1.11 (1.05, 1.18) <0.001	1 (Reference)	1.22 (1.14, 1.31) <0.001	1.24 (1.16, 1.33) <0.001	1.38 (1.25, 1.52) <0.001	0.044

Results was represented as HR (95%CI) *P*-value

a Crude model

b Model was adjusted with sex, age, race, educational level, TDI, physical activity, drinking status

c Model was further adjusted with hypertension, diabetes, health diet score base on model 2

**Table 2:** Subgroup analysis of association between maternal smoking and risk of stroke

Subgroup ^a^	N	HR (95%CI)	*P* for interaction
Sex			0.179
Male	143,020	1.08 (1.00, 1.16)	
Female	161,964	1.17 (1.07, 1.28)	
Diabetes			0.238
Yes	16,644	1.19 (1.02, 1.39)	
No	288,340	1.10 (1.04, 1.17)	
Hypertension			0.762
Elevated	38,743	1.11 (0.92, 1.34)	
Normal	48,716	1.05 (0.86, 1.29)	
Stage 1	83,014	1.18 (1.05, 1.32)	
Stage 2	134,511	1.10 (1.02, 1.18)	
Body mass index			0.403
>=25	199,911	1.10 (1.03, 1.17)	
< 25	105,073	1.16 (1.04, 1.30)	
Age			0.881
>=65	53,351	1.12 (1.02, 1.23)	
< 65	251,633	1.12 (1.05, 1.20)	

Results was represented as HR (95%CI) *P*-value

a Model was adjusted with sex, age, race, educational level, TDI, physical activity, drinking status, hypertension, diabetes and health diet score

**Table 3:** Subgroup analysis of association between age of smoking initiation and risk of stroke

Subgroup ^a^		HR (95%CI)	*P* for interaction
Sex			0.047
Male	< 15	1.32 (1.17, 1.47)	
	15–18	1.19 (1.09, 1.29)	
	>=18	1.16 (1.06, 1.27)	
Female	< 15	1.57 (1.28, 1.93)	
	15–18	1.36 (1.22, 1.52)	
	>=18	1.32 (1.19, 1.46)	
Diabetes			0.286
Yes	< 15	1.26 (1.00, 1.60)	
	15–18	1.16 (0.98, 1.38)	
	>=18	1.05 (0.87, 1.27)	
No	< 15	1.40 (1.26, 1.56)	
	15–18	1.26 (1.17, 1.35)	
	>=18	1.25 (1.17, 1.35)	
Hypertension			0.004
Elevated	< 15	1.17 (0.82, 1.67)	
	15–18	1.12 (0.89, 1.41)	
	>=18	1.19 (0.95, 1.49)	
Normal	< 15	1.69 (1.22, 2.35)	
	15–18	1.56 (1.25, 1.96)	
	>=18	1.38 (1.09, 1.75)	
Stage 1	< 15	1.17 (0.93, 1.46)	
	15–18	1.41 (1.23, 1.61)	
	>=18	1.15 (0.99, 1.33)	
Stage 2	< 15	1.45 (1.28, 1.64)	
	15–18	1.16 (1.07, 1.27)	
	>=18	1.24 (1.14, 1.35)	
Body mass index			0.017
>=25	< 15	1.35 (1.20, 1.50)	
	15–18	1.25 (1.16, 1.34)	
	>=18	1.17 (1.08, 1.27)	
< 25	< 15	1.48 (1.19, 1.85)	
	15–18	1.19 (1.04, 1.37)	
	>=18	1.36 (1.20, 1.55)	
Age			0.045
>=65	< 15	1.46 (1.23, 1.73)	
	15–18	1.27 (1.15, 1.41)	
	>=18	1.16 (1.04, 1.28)	
< 65	< 15	1.36 (1.20, 1.53)	
	15–18	1.24 (1.14, 1.35)	
	>=18	1.28 (1.18, 1.40)	

Results was represented as HR (95%CI) *P*-value

a Model was adjusted with sex, age, race, educational level, TDI, physical activity, drinking status, hypertension, diabetes and health diet score

**Table 4:** Accelerated biological ageing as a potential mediator mediates the association between early-life tobacco smoke exposure and risk of stroke

Mediator ^a, b^	Adulthood vs. Never smokers	Adolescence vs. Never smokers	Childhood vs. Never smokers
PhenoAge AA			
Direct Effects	0.004 (0.002–0.01)	0.003 (0.002–0.011)	0.004 (0.002–0.01)
Indirect Effects	0.0008 (0.0007–0.0009)	0.0009 (0.0008–0.001)	0.0014 (0.0012–0.0017)
Proportion of mediation	18.34%	21.70%	25.10%
KDM-BA AA			
Direct Effects	0.004 (0.002–0.01)	0.004 (0.002–0.01)	0.004 (0.002–0.01)
Indirect Effects	0.0002 (0.0002–0.0003)	0.0005 (0.0004–0.0006)	0.0008 (0.0007–0.001)
Proportion of mediation	6.44%	11.10%	16.64%

a Model was adjusted with sex, age, race, educational level, TDI, physical activity, drinking status, hypertension, diabetes and health diet score

b Results were presented as β (95%CI)

### Electronic supplementary material

Below is the link to the electronic supplementary material.

Supplementary Material 1

## Data Availability

All data used in this study can be found in UK Biobank https://www.ukbiobank.ac.uk/
