# Supplementary material for: Early-life tobacco smoke exposure and stroke risk: a prospective study of 341,783 and 352,737 UK Biobank participants
Source: BMC Public Health. 2024 May 17;24:1339. doi: 10.1186/s12889-024-18588-6 (PMC11102258; doi:10.1186/s12889-024-18588-6)

Table Page

Table S1. Details regarding the corresponding UK Biobank data sources in the algorithm for biological age

| Items in UK Biobank data dictionary | Field ID in UK Biobank |
| --- | --- |
| Forced expiratory volume in one second (FEV1) (L) | 3063 |
| Systolic blood pressure, automated reading (mmHg) | 4080 |
| Cholesterol (mg/dL) | 30690 |
| Glycated haemoglobin (HbA1c) (%) | 30750 |
| Urea (mg/dL) | 30670 |
| Lymphocyte percentage (%) | 30180 |
| Mean sphered cell volume (fL) | 30270 |
| Glucose (mg/dL) | 30740 |
| Red blood cell (erythrocyte) distribution width (%) | 30070 |
| White blood cell (leukocyte) count (1000cells/uL) | 30000 |
| Albumin (g/dL) | 30600 |
| Creatinine (mg/dL) | 30700 |
| C-reactive protein (mg/dL) | 30710 |
| Alkaline phosphatase (U/L) | 30610 |

Table S2. Baseline characteristics of the participants

|  |  |  | Age of smoking initiation | | | |  |  | In utero tobacco smoke exposure | |  |
| --- | --- | --- | --- | --- | --- | --- | --- | --- | --- | --- | --- |
|  |  | Overall | Never smokers | Adulthood >=18 years | Adolescence 15-18 years | Childhood < 15 years | P-Value | Overall | No | Yes | P-Value |
| Number |  | 342893 | 233792 | 16969 | 46612 | 45520 |  | 304984 | 215730 | 89254 |  |
| Age (mean (SD)) |  | 56.28 (8.10) | 55.79 (8.15) | 55.57 (7.99) | 57.55 (7.69) | 57.81 (7.97) | <0.001 | 56.06 (8.13) | 56.17 (8.28) | 55.80 (7.77) | <0.001 |
| Sex (%) | Female | 181601 (53.0) | 132747 (56.8) | 5808 (34.2) | 19896 (42.7) | 23150 (50.9) | <0.001 | 161964 (53.1) | 115725 (53.6) | 46239 (51.8) | <0.001 |
|  | Male | 161292 (47.0) | 101045 (43.2) | 11161 (65.8) | 26716 (57.3) | 22370 (49.1) |  | 143020 (46.9) | 100005 (46.4) | 43015 (48.2) | |
| Race (%) | Asia | 5677 (1.7) | 4938 (2.1) | 76 (0.4) | 161 (0.3) | 502 (1.1) | <0.001 | 5673 (1.9) | 5530 (2.6) | 143 (0.2) | <0.001 |
|  | Black | 1964 (0.6) | 1722 (0.7) | 23 (0.1) | 60 (0.1) | 159 (0.3) |  | 1923 (0.6) | 1890 (0.9) | 33 (0.0) | |
|  | Other | 20498 (6.0) | 13936 (6.0) | 849 (5.0) | 2137 (4.6) | 3576 (7.9) |  | 19047 (6.2) | 15732 (7.3) | 3315 (3.7) | |
|  | White | 314754 (91.8) | 213196 (91.2) | 16021 (94.4) | 44254 (94.9) | 41283 (90.7) |  | 278341 (91.3) | 192578 (89.3) | 85763 (96.1) | |
| Educational level (%) | Below college or vocational education | 125333 (36.6) | 79163 (33.9) | 8572 (50.5) | 22137 (47.5) | 15461 (34.0) | <0.001 | 109336 (35.8) | 73460 (34.1) | 35876 (40.2) | <0.001 |
|  | College or vocational education | 217560 (63.4) | 154629 (66.1) | 8397 (49.5) | 24475 (52.5) | 30059 (66.0) |  | 195648 (64.2) | 142270 (65.9) | 53378 (59.8) | |
| BMI (mean (SD)) |  | 27.24 (4.66) | 26.98 (4.61) | 28.53 (5.02) | 27.86 (4.58) | 27.46 (4.69) | <0.001 | 27.21 (4.65) | 26.98 (4.55) | 27.76 (4.84) | <0.001 |
| TDI (mean (SD)) |  | -1.46 (2.98) | -1.70 (2.85) | -0.40 (3.36) | -1.09 (3.13) | -1.02 (3.16) | <0.001 | -1.47 (2.98) | -1.53 (2.96) | -1.31 (3.03) | <0.001 |
| Metabolic equivalent of task (mean (SD)) | | 2651.11 (2696.58) | 2622.06 (2624.59) | 2975.89 (3259.96) | 2761.29 (2878.09) | 2566.45 (2624.70) | <0.001 | 2652.40 (2693.51) | 2615.31 (2637.66) | 2742.07 (2821.93) | <0.001 |
| Drinking status (%) | Current | 318335 (92.8) | 215805 (92.3) | 15675 (92.4) | 43957 (94.3) | 42898 (94.2) | <0.001 | 282986 (92.8) | 199197 (92.3) | 83789 (93.9) | <0.001 |
|  | Never | 13196 (3.8) | 11552 (4.9) | 248 (1.5) | 636 (1.4) | 760 (1.7) |  | 12142 (4.0) | 9909 (4.6) | 2233 (2.5) | |
|  | Previous | 11362 (3.3) | 6435 (2.8) | 1046 (6.2) | 2019 (4.3) | 1862 (4.1) |  | 9856 (3.2) | 6624 (3.1) | 3232 (3.6) | |
| Hypertension (%) | elevated | 43184 (12.6) | 30085 (12.9) | 2026 (11.9) | 5480 (11.8) | 5593 (12.3) | <0.001 | 38743 (12.7) | 27400 (12.7) | 11343 (12.7) | <0.001 |
|  | normal | 53516 (15.6) | 38033 (16.3) | 2494 (14.7) | 6317 (13.6) | 6672 (14.7) |  | 48716 (16.0) | 35142 (16.3) | 13574 (15.2) | |
|  | stage1 | 92894 (27.1) | 63877 (27.3) | 4635 (27.3) | 12292 (26.4) | 12090 (26.6) |  | 83014 (27.2) | 58420 (27.1) | 24594 (27.6) | |
|  | stage2 | 153299 (44.7) | 101797 (43.5) | 7814 (46.0) | 22523 (48.3) | 21165 (46.5) |  | 134511 (44.1) | 94768 (43.9) | 39743 (44.5) | |
| Diabetes (%) | No | 323835 (94.4) | 222981 (95.4) | 15397 (90.7) | 43058 (92.4) | 42399 (93.1) | <0.001 | 288340 (94.5) | 204271 (94.7) | 84069 (94.2) | <0.001 |
|  | Yes | 19058 (5.6) | 10811 (4.6) | 1572 (9.3) | 3554 (7.6) | 3121 (6.9) |  | 16644 (5.5) | 11459 (5.3) | 5185 (5.8) | |
| Health diet score (%) | Low | 39976 (11.7) | 24336 (10.4) | 3283 (19.3) | 6877 (14.8) | 5480 (12.0) | <0.001 | 35345 (11.6) | 23796 (11.0) | 11549 (12.9) | <0.001 |
|  | Mid | 165672 (48.3) | 110902 (47.4) | 8940 (52.7) | 23840 (51.1) | 21990 (48.3) |  | 147066 (48.2) | 103151 (47.8) | 43915 (49.2) | |
|  | High | 137245 (40.0) | 98554 (42.2) | 4746 (28.0) | 15895 (34.1) | 18050 (39.7) |  | 122573 (40.2) | 88783 (41.2) | 33790 (37.9) | |
| Stroke (%) | No | 336101 (98.0) | 229838 (98.3) | 16503 (97.3) | 45380 (97.4) | 44380 (97.5) | <0.001 | 299093 (98.1) | 211669 (98.1) | 87424 (97.9) | 0.002 |
|  | Yes | 6792 (2.0) | 3954 (1.7) | 466 (2.7) | 1232 (2.6) | 1140 (2.5) |  | 5891 (1.9) | 4061 (1.9) | 1830 (2.1) | |
| LE8 (mean (SD)) |  | 67.07 (11.67) | 70.02 (10.68) | 58.98 (11.63) | 60.85 (11.01) | 61.33 (11.14) | <0.001 | 67.07 (11.71) | 67.52 (11.61) | 65.98 (11.88) | <0.001 |
| KDM-BA AA (mean (SD)) |  | 0.00 (1.00) | -0.04 (0.98) | 0.18 (1.11) | 0.06 (1.02) | 0.01 (1.00) | <0.001 | 0.00 (1.00) | -0.02 (1.00) | 0.02 (0.99) | <0.001 |
| PhenoAge AA (mean (SD)) |  | 0.00 (1.00) | -0.11 (0.96) | 0.39 (1.06) | 0.23 (1.03) | 0.14 (1.03) | <0.001 | 0.00 (1.00) | -0.05 (0.99) | 0.06 (1.01) | <0.001 |

BMI: body mass index; TDI: Townsend Deprivation Index; AA: Age-accelerated

The results of the data from a normal distribution are presented as Mean (SD)

Table S3. Subgroup analysis of association between exposure to tobacco smoke during early life and risk of stroke based on maternal smoking status.

| In utero tobacco smoke exposure ^a^ | Never smokers | Adulthood ≥ 18 years | Adolescence 15-18 years | Childhood < 15 years |
| --- | --- | --- | --- | --- |
|  |  | HR(95%CI) P-value | HR(95%CI) P-value | HR(95%CI) P-value |
| Yes | 1 (Reference) | 1.24 (1.08, 1.42) 0.002 | 1.23 (1.09, 1.39) 0.001 | 1.42 (1.21, 1.67) <0.001 |
| No | 1 (Reference) | 1.20 (1.10, 1.31) <0.001 | 1.20 (1.10, 1.31) <0.001 | 1.30 (1.12, 1.51) <0.001 |

Results was represented as HR (95%CI) P-value

a Model was adjusted with sex, age, race, educational level, TDI, physical activity, drinking status, hypertension, diabetes and health diet score

Table S4. Association between biological ageing and age at smoking initiation

|  | Never smokers | Adulthood ≥ 18 years | Adolescence 15-18 years | Childhood < 15 years | Continuous |
| --- | --- | --- | --- | --- | --- |
|  |  | Beta (95%CI) P-Value | Beta (95%CI) P-Value | Beta (95%CI) P-Value |  |
| PhenoAge AA | 1 (Reference) | 1.21 (1.20, 1.23) <0.001 | 1.26 (1.24, 1.27) <0.001 | 1.39 (1.36, 1.41) <0.001 | <0.001 |
| KDM AA | 1 (Reference) | 1.06 (1.05, 1.07) <0.001 | 1.12 (1.11, 1.14) <0.001 | 1.24 (1.22, 1.26) <0.001 | <0.001 |

Results was represented as HR (95%CI) P-value

Model was adjusted with sex, age, race, educational level, TDI, physical activity, drinking status, hypertension, diabetes and health diet score

Table S5. Sensitivity analysis excluding participants who developed stroke within one year of follow-up

|  | In utero tobacco smoke exposure | | Age of smoking initiation |  |  |  |
| --- | --- | --- | --- | --- | --- | --- |
|  | No | Yes | Never smokers | Adulthood ≥ 18 years | Adolescence 15-18 years | Childhood < 15 years |
|  |  | HR (95%CI) P-Value | HR (95%CI) P-Value | HR (95%CI) P-Value | HR (95%CI) P-Value | HR (95%CI) P-Value |
| Model 1 ^a^ | 1 (Reference) | 1.09 (1.03, 1.15) 0.004 | 1 (Reference) | 1.49 (1.39, 1.60) <0.001 | 1.56 (1.45, 1.67) <0.001 | 1.65 (1.48, 1.83) <0.001 |
| Model 2 ^b^ | 1 (Reference) | 1.12 (1.05, 1.18) <0.001 | 1 (Reference) | 1.22 (1.13, 1.31) <0.001 | 1.23 (1.14, 1.32) <0.001 | 1.39 (1.25, 1.55) <0.001 |
| Model 3 ^c^ | 1 (Reference) | 1.11 (1.05, 1.18) <0.001 | 1 (Reference) | 1.21 (1.12, 1.30) <0.001 | 1.20 (1.12, 1.30) <0.001 | 1.36 (1.22, 1.52) <0.001 |

Results was represented as HR (95%CI) P-value

a Crude model

b Model was adjusted with sex, age, race, educational level, TDI, physical activity, drinking status

c Model was further adjusted with hypertension, diabetes, health diet score base on model 2

Table S6. Sensitivity analysis excluding participants with diabetes

|  | In utero tobacco smoke exposure | | Age of smoking initiation |  |  |  |
| --- | --- | --- | --- | --- | --- | --- |
|  | No | Yes | Never smokers | Adulthood ≥ 18 years | Adolescence 15-18 years | Childhood < 15 years |
|  |  | HR (95%CI) P-Value | HR (95%CI) P-Value | HR (95%CI) P-Value | HR (95%CI) P-Value | HR (95%CI) P-Value |
| Model 1 ^a^ | 1 (Reference) | 1.07 (1.01, 1.13) 0.028 | 1 (Reference) | 1.52 (1.42, 1.62) <0.001 | 1.61 (1.51, 1.71) <0.001 | 1.69 (1.53, 1.86) <0.001 |
| Model 2 ^b^ | 1 (Reference) | 1.10 (1.04, 1.17) 0.001 | 1 (Reference) | 1.25 (1.17, 1.35) <0.001 | 1.26 (1.18, 1.36) <0.001 | 1.41 (1.26, 1.57) <0.001 |
| Model 3 ^c^ | 1 (Reference) | 1.10 (1.04, 1.17) 0.001 | 1 (Reference) | 1.25 (1.17, 1.35) <0.001 | 1.26 (1.17, 1.35) <0.001 | 1.40 (1.26, 1.56) <0.001 |

Results was represented as HR (95%CI) P-value

a Crude model

b Model was adjusted with sex, age, race, educational level, TDI, physical activity, drinking status

c Model was further adjusted with hypertension, diabetes, health diet score base on model 2

Table S7. Sensitivity analysis using one complete sample and adjust further smoking related covariates.

| In utero tobacco smoke exposure ^a^ | | Age of smoke initiation ^b^ | |  |  |  |
| --- | --- | --- | --- | --- | --- | --- |
| No | Yes | Never smokers | Adulthood ≥18 years | Adolescence 15-18 years | Childhood <15 years | P for trend |
|  | HR (95%CI) P-Value |  | HR (95%CI) P-Value | HR (95%CI) P-Value | HR (95%CI) P-Value |  |
| 1 (Reference) | 1.10 (1.04, 1.16) 0.001 | 1 (Reference) | 1.21 (1.13, 1.30) <0.001 | 1.21 (1.13, 1.30) <0.001 | 1.35 (1.21, 1.51) <0.001 | 0.036 |

a Model was adjusted for age, sex, race, educational level, TDI, physical activity, drinking status, hypertension, diabetes, health diet scores and age of smoke initiation

b Model was adjusted for age, sex, race, educational level, TDI, physical activity, drinking status, hypertension, diabetes, health diet scores and in utero tobacco smoke exposure

Table S8. Sensitivity analysis excluding participants with home smokers.

| Age of smoke initiation ^a^ | |  |  |  |
| --- | --- | --- | --- | --- |
| Never smokers | Adulthood ≥18 years | Adolescence 15-18 years | Childhood <15 years | P for trend |
|  | HR (95%CI) P-Value | HR (95%CI) P-Value | HR (95%CI) P-Value |  |
| 1 (Reference) | 1.80 [1.48, 2.20] <0.001 | 1.60 [1.36, 1.89] <0.001 | 1.56 [1.33, 1.85] <0.001 | 0.001 |

a Model was adjusted for age, sex, race, educational level, TDI, physical activity, drinking status, hypertension, diabetes, and health diet scores

Table S9. Sensitivity analysis after further adjusted stop-smoking status to the model.

| Age of smoke initiation ^a^ | |  |  |  |
| --- | --- | --- | --- | --- |
| Never smokers | Adulthood ≥18 years | Adolescence 15-18 years | Childhood <15 years | P for trend |
|  | HR (95%CI) P-Value | HR (95%CI) P-Value | HR (95%CI) P-Value |  |
| 1 (Reference) | 2.09 [1.84, 2.37] | 1.93 [1.74, 2.13] | 1.90 [1.72, 2.10] | 0.001 |

a Model was adjusted for age, sex, race, educational level, TDI, physical activity, drinking status, hypertension, diabetes, health diet scores and ever stop smoking for 6 months

Figure Page

Figure S1. Selection process of the study population


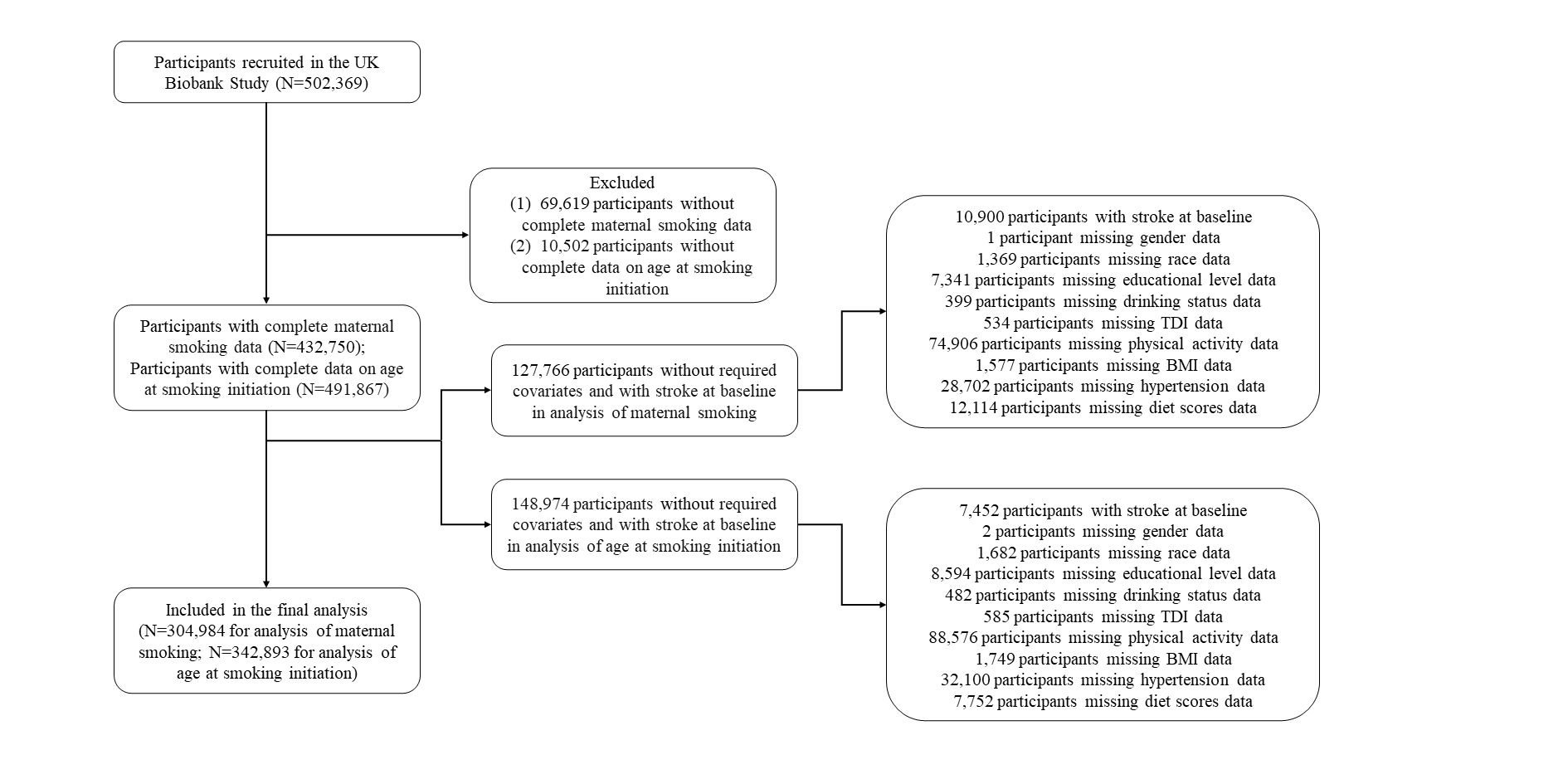

Supplement: Supplementary file 1 — Supplementary Material 1 [file 12889_2024_18588_MOESM1_ESM.docx]
